# Rethinking pattern hair loss classification in the era of trichoscopy and artificial intelligence

**DOI:** 10.3389/fmed.2026.1751134

**Published:** 2026-03-10

**Authors:** Luis E. Sánchez-Dueñas, Mariana León Quintero-Loreto, Jessica A. Moreno-Alanis, Deyanira G. Quiñones-Hernández, Mariana Larios-Cárdenas

**Affiliations:** Dermatologic Institute of Jalisco “Dr. José Barba Rubio”, University of Guadalajara, Jalisco, Mexico

**Keywords:** artificial intelligence in trichology, digital imaging assessment, hair loss classification, pattern hair loss evaluation, *trichoscopy*

## 1 Introduction

For decades, clinical classification systems have been central to the assessment of pattern hair loss, providing a shared framework for diagnosis and communication. Foundational scales, such as Hamilton's, based on observations of more than 700 individuals across age, sex, and ethnicity (1), and Norwood's refinement of these patterns through the study of over 1,000 Caucasian men (1, 2), established the basis for modern staging. Additional systems, including the Ludwig and Sinclair scales for women and the Basic and Specific classification, further sought to balance clinical detail with practical reproducibility (1, 2).

Despite their value, these systems show significant limitations in modern practice. Reproducibility varies widely, with lower agreement reported for Norwood-Hamilton staging compared with the Basic and Specific system (3), and important clinical presentations such as isolated vertex thinning and ethnic variation often fall outside their structure (4). No existing scale fully satisfies the criteria of accuracy, reproducibility, universality, and clinical applicability (1).

As trichology advances, there is a growing need for objective and scalable tools capable of detecting early or subtle changes that visual staging may miss. Digital imaging, trichoscopy, and other non-invasive quantitative methods now offer greater measurement fidelity than traditional visual scales (5). With progress in artificial intelligence and high-resolution imaging, the field is moving toward data-driven, technology-enabled classification systems. While traditional classification systems have effectively guided clinical understanding for decades, the advent of novel therapies for androgenetic alopecia and critical advances in trichoscopic and AI-enabled diagnostic and monitoring technologies compel a re-examination of disease classification. In this Opinion, we argue that these tools should be systematically adopted in research settings to generate standardized, biologically informed datasets that can support the evolution of future classification frameworks.

## 2 Current challenges and advances in pattern hair loss classification

### 2.1 Strengths and historical importance of traditional classification Systems

Traditional pattern hair loss classification systems have shaped diagnostic and therapeutic practice for many decades, serving as foundational tools for both clinicians and patients. Widely adopted frameworks such as the Norwood-Hamilton and Ludwig systems have long guided the identification of clinical stage, supported the evaluation of treatment response, and assisted practitioners in selecting appropriate surgical strategies when needed (2). Their strength lies in their simplicity and accessibility, which allow clinicians to rapidly categorize hair loss patterns in everyday practice without specialized equipment or advanced imaging.

Beyond clinical utility, composite clinical scales strengthen research by combining several observable features into a single measure, helping minimize the statistical difficulties that arise when individual traits interact in complex ways (6). Health measurement scales have become essential components of clinical medicine more broadly, allowing clinicians to assess traits that cannot be directly measured and to guide intervention planning and public health system management (6). Their long-standing use in epidemiologic studies reflects their practicality and the ongoing need for classifications that balance detail with reproducibility to support reliable monitoring of disease progression (1). Although many scales exist, each with its limitations, their continued relevance in everyday practice underscores their enduring clinical utility (2).

### 2.2 Limitations in accuracy and reproducibility

Despite their historical relevance, traditional classification systems show important weaknesses in accuracy and reproducibility because they rely on subjective visual interpretation. Controlled reliability studies have repeatedly demonstrated that even experienced dermatologists struggle to score the same images consistently. When seven dermatologists and 16 dermatology residents classified a set of clinical photographs, the inter-rater agreement yielded intra-class correlation coefficients ranging from 0.63 to 0.68, indicating unsatisfactory concordance (7).

The design of most traditional scales further contributes to these limitations. Existing methods for male- and female-pattern hair loss primarily describe surface-level patterns and do not incorporate trichoscopic markers or biological parameters that reflect disease progression (8). Wide spacing between categories makes it difficult to capture early changes, and the lack of objective criteria limits their ability to reflect the disease's actual trajectory over time (8). Comparative research also shows that reproducibility varies significantly across scales, with the Basic and Specific system demonstrating higher agreement and repeatability. In contrast, the Norwood-Hamilton system consistently shows the lowest match rates (3). Ultimately, traditional scales do not meet the standards of objectivity required for precise monitoring or early detection, limiting their role as fully reliable diagnostic instruments (9).

### 2.3 The gap between traditional scales and modern trichoscopic findings

Modern trichology increasingly recognizes that traditional visual staging systems cannot capture microscopic and quantitative features that reveal the true biology of hair loss. Trichoscopy provides objective, high resolution information about hair shaft diameter, follicular density, follicular units, and early signs of miniaturization (8). Automated image analysis systems such as TrichoScan quantify terminal and miniaturized hair density, anagen and telogen ratios, and hair caliber proportions, with operator agreement levels of approximately 97%, making them highly reliable for detecting early changes (5). Trichoscopic studies consistently document hallmark indicators such as hair diameter variability, increased vellus hairs, and the peripilar sign, which may be present long before a patient reaches a higher visual stage (10).

However, despite these advances, the integration of trichoscopic and AI-based metrics into a coherent classification framework remains limited. Current tools rely on heterogeneous measurement protocols, proprietary software platforms, and variable definitions of key parameters, resulting in inconsistent reporting across studies and limited comparability between datasets (8, 11). The literature reflects substantial variability in which metrics are measured, how they are calculated, and how thresholds are interpreted, underscoring the absence of standardized reference values and consensus on clinical relevance. As artificial intelligence–based systems continue to improve in accuracy and scalability, this lack of harmonization increasingly represents a methodological bottleneck rather than a technological one (12). Addressing this gap will require systematic, research-focused efforts to validate, prioritize, and standardize trichoscopic and AI-derived metrics. Without this foundational work, advances in imaging and computation risk remaining fragmented and underutilized rather than transformative.

### 2.4 Emerging digital and artificial intelligence-based approaches

**Figure 1:**
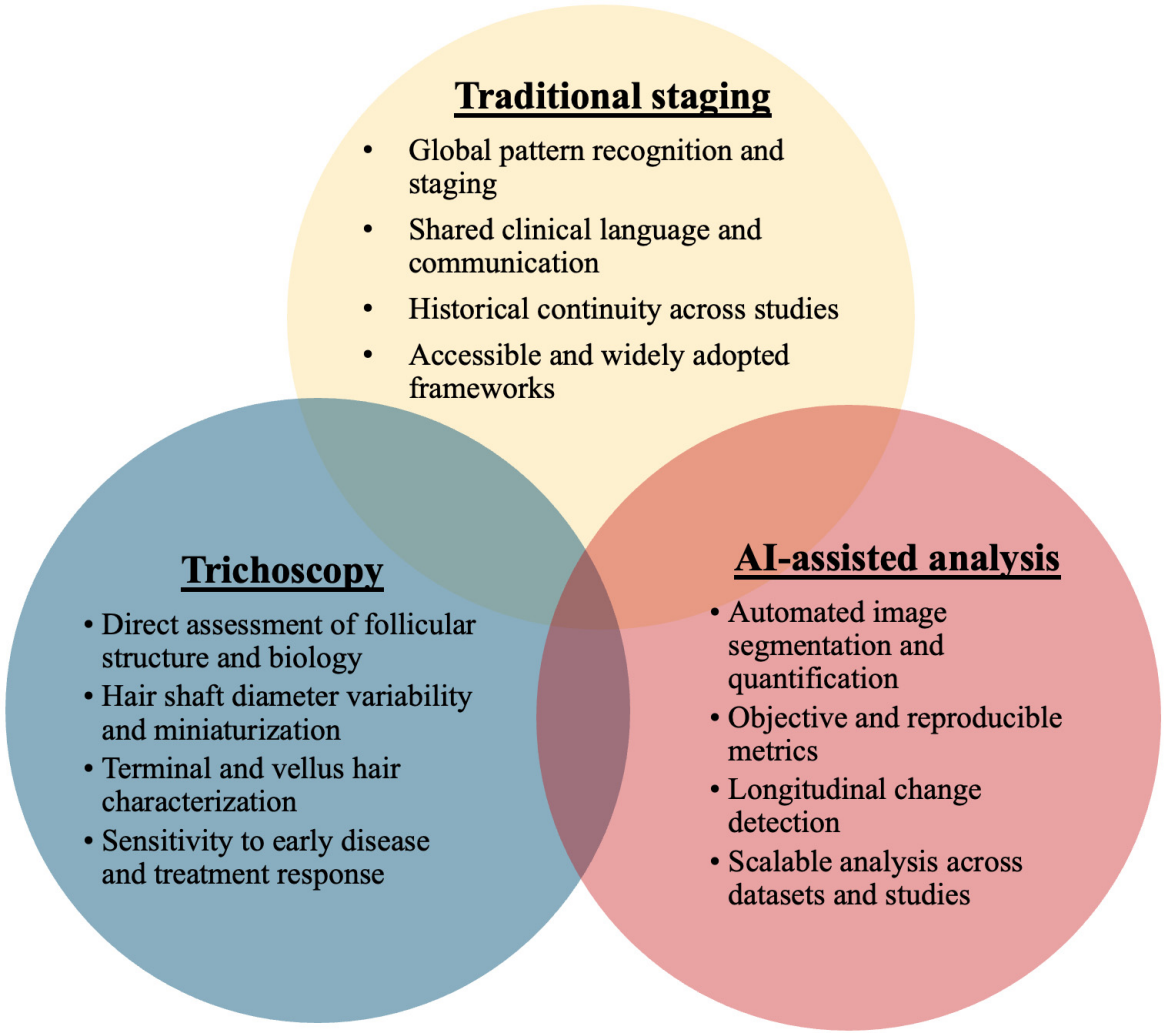
A hybrid research framework for pattern hair loss classification.

Advances in artificial intelligence have introduced new classification systems that offer greater precision and objectivity than traditional visual staging. Artificial intelligence-supported frameworks can automatically identify hair loss regions, quantify surface involvement, and compute standardized metrics such as area ratios, providing a more reliable alternative to length-based measurements used in older scales (12). Automated image analysis also detects subtle changes in hair alignment and volume that human observers may overlook, particularly when evaluating two dimensional images (13). These developments parallel findings from broader dermatology, where artificial intelligence systems already achieve diagnostic accuracy comparable to that of dermatologists, underscoring their potential relevance to trichology (14). In addition, integrating artificial intelligence with trichoscopy enables quantification of growth patterns, monitoring of therapeutic response, and incorporation of physiologic and microbiologic parameters into a more comprehensive evaluation of scalp health (15). Figure 1 illustrates the complementary roles of traditional pattern classification, trichoscopy, and artificial intelligence–assisted analysis within a research-driven classification framework.

Despite these advances, substantial methodological and practical limitations constrain the current role of artificial intelligence in hair-loss classification. Existing models may fail to detect very small or early areas of pattern hair loss, reducing sensitivity, and frequently struggle with intermediate stages where phenotypic boundaries overlap rather than conform to discrete categories (12). Performance is further influenced by variability in image acquisition, including differences in lighting, camera systems, magnification, and viewing angle, reflecting the absence of standardized imaging protocols in dermatology comparable to those established in radiology (14). Beyond imaging heterogeneity, there is no consensus regarding which quantitative metrics should be measured, how they should be calculated, or how thresholds should be interpreted across different trichoscopic and imaging platforms, limiting reproducibility and cross-study comparability. Addressing these challenges will require coordinated, research-driven efforts focused on dataset curation, harmonized acquisition protocols, and validation of AI-derived metrics. Only through this evidence-building phase can artificial intelligence meaningfully inform classification systems rather than simply replicate existing visual scales in digital form.

## 3 Discussion

The limitations of traditional visual scales and rapid advances in digital imaging and computational analysis underscore the need for an integrated, future-oriented assessment strategy. From a research perspective, this moment represents an opportunity to rethink how classification systems are built, validated, and refined using objective, quantitative data. As modern trichology increasingly relies on quantitative metrics and high-resolution imaging, hybrid models offer a path toward a more complete, biologically accurate evaluation. Recent work combining deep learning-based image segmentation with quantitative surface-area measurements demonstrates how objective computational outputs can overcome the coarse granularity of classical classifications (12). These approaches generate reproducible, fine-grained grading systems that better align with the complexity of hair loss biology and provide a more accurate basis for monitoring therapeutic response.

A hybrid model also reflects the expanding role of machine learning in pattern hair loss. The development of scalp dermoscopy image databases, automated classification tools, and predictive models for disease progression will require coordinated effort from hair specialists, particularly in the creation of standardized, high-quality annotated datasets (16). By integrating these computational resources with routine trichoscopic evaluation, clinicians can enhance both diagnostic precision and longitudinal monitoring. Trichoscopy remains indispensable because it captures structural follicular changes that correspond directly to biological processes. When performed by experienced clinicians, trichoscopy correlates strongly with automated measures of terminal, vellus, and total hair counts, reinforcing its role as a sensitive and biologically grounded tool for treatment assessment (17). Even for less experienced practitioners, focusing on the most robust quantitative indicators allows trichoscopy to reliably support follow-up and treatment evaluation (17).

Taken together, these advancements point toward a next-generation assessment model that blends three complementary pillars: traditional pattern recognition for global staging, trichoscopy for detailed structural metrics, and artificial intelligence–assisted analysis for standardized and reproducible quantification. Integrating these approaches addresses the limitations of each method when used in isolation and positions modern trichology to adopt a more precise, biologically informed, and technology-enabled framework for evaluating hair loss. Such a hybrid model represents a necessary evolution beyond purely visual classification. In our view, its primary value lies in research, where the systematic integration of traditional staging, trichoscopy, and artificial intelligence can generate the evidence base required to responsibly advance classification systems and, ultimately, inform future clinical practice.
